# Topographical control of cell-cell interaction in C6 glioma by nanodot arrays

**DOI:** 10.1186/1556-276X-9-250

**Published:** 2014-05-21

**Authors:** Chia-Hui Lee, Ya-Wen Cheng, G Steven Huang

**Affiliations:** 1 Department of Materials Science and Engineering, National Chiao Tung University, 1001 University Road, Hsinchu 300, Taiwan; 2 Institute of Biomedical Engineering, National Chiao Tung University, 1001 University Road, Hsinchu 300, Taiwan

**Keywords:** Nanotopography, Cell networking, Cell-cell communications, Neuron

## Abstract

Nanotopography modulates the physiological behavior of cells and cell-cell interactions, but the manner of communication remains unclear. Cell networking (syncytium) of astroglia provides the optimal microenvironment for communication of the nervous system. C6 glioma cells were seeded on nanodot arrays with dot diameters ranging from 10 to 200 nm. Cell viability, morphology, cytoskeleton, and adhesion showed optimal cell growth on 50-nm nanodots if sufficient incubation was allowed. In particular, the astrocytic syncytium level maximized at 50 nm. The gap junction protein Cx43 showed size-dependent and time-dependent transport from the nucleus to the cell membrane. The transport efficiency was greatly enhanced by incubation on 50-nm nanodots. In summary, nanotopography is capable of modulating cell behavior and influencing the cell-cell interactions of astrocytes. By fine-tuning the nanoenvironment, it may be possible to regulate cell-cell communications and optimize the biocompatibility of neural implants.

## Background

Astrocytes, also known collectively as astroglia, are characteristic star-shaped glial cells in the brain and spinal cord. Astrocytes are the most abundant cells in the human brain. They perform many functions, including biochemical support of the endothelial cells that form the blood-brain barrier, provision of nutrients to nervous tissue, and maintenance of extracellular ion balance. Additionally, astrocytes play a role in the repair and scarring process of the brain and spinal cord following traumatic injuries.

Reproducing the complexity of the astrocytic syncytium (cell network) to support neuron regeneration in the brain is a major topic in neuroscience research. The astrocytic syncytium is considered a structural support for neurons with respect to cell-to-cell signaling. In addition to cell contact-mediated communication, in which small molecules pass through intercellular channels, astrocytes also communicate using extracellular signaling pathways and networks in a chain reaction. Astrocyte-astrocyte and astrocyte-neuron communication occurs primarily through chemical signals [1]. The local microenvironment regulates neuronal regeneration through the astrocytic syncytium.

Micro- and nanotographic environments affect cell growth, adhesion, and physiological functions. Astroglial cells had much better cell spreading and adhesion when grown on larger micro-pillar spacing [2,3]. Microgroove structures controlled the growth pattern in C6 glioma cells [4] and upregulated the expression levels of communication-related proteins such as the connexin family in neurons [5]. Nanopost surfaces enhanced focal adhesions in endothelial cells [6] and elongated the cell body of fibroblasts [7]. It has been demonstrated that neurons are sensitive to topographic cues of 10 nm [8]. Nanoscale structures interact with cells and direct cellular growth through mechanisms that might be different from those of microscale structures [9].

Nanotopography regulates and guides the astrocytic syncytium. A 200-nm ridge/groove structure affected the morphology and alignment of C6 glioma cells [4]. Multi-walled carbon nanotube (CNT) arrays with chemical modifications and 3D nanotopography greatly enhanced the adhesion and organization of the functional neuronal network [10,11]. Positively charged nanofibers dictated neuron adhesion and network formation [12]. CNT clusters promoted complex and interconnected neuronal network formation via the self-assembly process of neurons [13,14]. Topography affects the growth direction of processes and the adhesion of astrocytes. Nanotopography might affect the constructs and functions of astrocytes, leading to the regulation of hyperexcitability and epileptic activity in neurons.

Structures with topographic patterns can control cell behavior, and the interactions between cells and substrates may play an important role in substrate biocompatibility [15]. However, the effects of glial-substrate interactions on the astrocytic syncytium are not clear. In this report, we used ordered nanotopography to study the molecular mechanisms underlying topographic control of the astrocytic syncytium of the C6 glioma. Nanotopography is capable of modulating transport of gap junction protein and influencing the cell-cell interactions of astrocytes.

## Methods

### Cell culture

The C6 glioma-astrocytoma rat cell line, C6.51.passage, was purchased from the Bioresource Collection and Research Center (BCRC; Hsinchu, Taiwan). C6 cells were cultured in Hamćs F10 medium with sodium bicarbonate (NaHCO_3_), horse serum (HS), fetal bovine serum (FBS), GlutaMAX I (Thermo Fisher Scientific Inc., Waltham, MA, USA), trypsin, and BSA (bovine serum albumin), which were purchased from GIBCO (Thermo Fisher Scientific Inc.). The cells were incubated at 37°C in 5% CO_2_.

### Chemicals

A CellTiter 96® AQueous One Solution Cell Proliferation Assay (MTS assay) was purchased from Promega (Madison, WI, USA). Phosphate-buffered saline (PBS) was purchased from Bio-tech (Taipei, Taiwan). Anti-vinculin antibody (vinculin) and anti-connexin43 antibody (connexin43) were purchased from Abcam (Cambridge, England, UK) and Invitrogen (Renfrew, UK), respectively. Anti-glial fibrillary acidic protein antibody (GFAP), luminol reagent, and oxidizing reagent were purchased from Millipore (Billerica, MA, USA). Sulfuric acid (H_2_SO_4_), oxalic acid (H_2_C_2_O_4_), and phosphoric acid (H_3_PO_4_) were purchased from Sigma Chemicals (Perth, Western Australia). Other chemicals of analytical grade or higher were purchased from Sigma or Millipore.

### Fabrication of nanodot surfaces

Nanodot arrays were fabricated as previously described [16]. A 200-nm-thick tantalum nitride (TaN) thin film was sputtered onto a 6-in silicon wafer (Summit-Tech, West Hartford, CT, USA), followed by a deposition of a 400-nm-thick aluminum (Admat-Midas, Norristown, PA, USA) layer on top of the TaN thin film. Anodization was performed using either 1.8 M H_2_SO_4_ at 5 V for 1.5 h (for the 10-nm nanodot array) or 0.3 M H_2_C_2_O_4_ at 25, 40, or 100 V for the 50-, 100-, and 200-nm nanodot arrays, respectively. Porous anodic alumina was formed during the anodic oxidation. The underlying TaN layer was oxidized into tantalum oxide nanodots using the alumina nanopores as a template. The porous alumina was then removed by immersing the array in 5% (*w*/*v*) H_3_PO_4_ for 6 h. The dimensions and homogeneity of the nanodot arrays were measured and calculated from images taken using a JEOL JSM-6500 thermal field emitter (TFE)-scanning electron microscope (SEM) (Tokyo, Japan).

### CellTiter 96® AQueous One Solution Cell Viability Assay

Cell viability was assessed using an MTS assay. All of the operational methods followed the Promega operation manual. The absorbance of the formazan product at 490 nm was measured directly from 96-well plates. A standard curve was generated with C6 astrocytes. The results were expressed as the mean ± SD of six experiments.

### Morphological observation by scanning electron microscopy

The C6 glioma cells were seeded on the different nanodot surfaces at a density of 5.0 × 10^3^ cells/cm^2^ for 24, 72, and 120 h of incubation. After removing the culture medium, the surfaces were rinsed three times with PBS. The cells were fixed with 1.25% glutaraldehyde in PBS at room temperature for 20 min, followed by post-fixation in 1% osmium tetroxide for 30 min. Dehydration was performed by 10-min incubation in each of a graded series of ethanol concentrations (40%, 50%, 60%, 70%, 75%, 80%, 85%, 90%, 95%, and 100%); after which, the samples were air dried. The specimens were sputter-coated with platinum and examined with a JEOL JSM-6500 TFE-SEM at an accelerating voltage of 5 kiloelectron volts (keV). The astrocytic syncytium level of the cells grown on the nanodots was quantified using ImageJ software and compared to the surface area of cells grown on a flat surface. The SEM images of six different substrate fields were measured per sample, and three separate samples were measured for each nanopore surface.

### Connexin43, GFAP, and vinculin immunostaining

The C6 glioma cells were seeded on the different nanodot surfaces at a density of 1.0 × 10^3^ cells/cm^2^ for 24, 72, and 120 h of incubation. The adhered cells were fixed with 4% paraformaldehyde (J.T. Baker, Center Valley, PA, USA) in PBS for 20 min followed by three washes with PBS. The cell membranes were permeabilized by incubating in 0.1% Triton X-100 for 10 min, followed by three PBS washes and blocking with 1% BSA in PBS for at 4°C overnight, followed by an additional three PBS washes. The samples were incubated overnight at 4°C with anti-connexin43, anti-GFAP, and anti-vinculin antibodies diluted in 1% BSA, followed by incubation with Alexa Fluor 488 goat anti-mouse and Alexa Fluor 532 goat anti-rabbit antibodies (Thermo Fisher Scientific) for 1.5 h, three PBS washes, and examination using a Leica TCS SP2 confocal microscope (Milton Keynes, UK). The connexin43 plaques, GFAP, and vinculin plaques per cell were determined by ImageJ.

### Western blotting

Cultured C6 glioma cells were lysed and centrifuged at 12,000 × *g* for 10 min at 4°C. The supernatants were transferred to new Eppendorf tubes (Hamburg, Germany), and the protein concentrations were determined by UV/vis spectroscopy. After the protein concentrations were determined, the supernatants were mixed with 4X sample buffer and lysis buffer to a final concentration of 1 mg/mL protein. The samples were heated at 95°C for 3 min and cooled at 0°C for 3 min; these steps were repeated three times. Proteins were separated using 10% SDS-PAGE gels and transferred to PVDF membranes. Nonspecific protein binding was blocked using a 5% milk solution at 4°C overnight. The membranes were subsequently blotted at 4°C overnight with the anti-connexin43 (Cx43) and GAPDH antibodies indicated for each experiment, which were diluted in blocking buffer. Specific primary antibodies were blotted using secondary antibodies in the blocking buffer at room temperature for 2 h. Chemiluminescence detection was performed using western blotting luminol and oxidizing reagents (Bio-Rad, CA, USA).

### Statistics

The means and standard deviations were calculated for the recorded data. Student's *t* test was employed to determine significant differences among the data sets, and significance was defined as a *p* value <0.05.

## Results and discussion

### Nanodot arrays modulated the cell viability of C6 glioma cells

**Figure 1:**
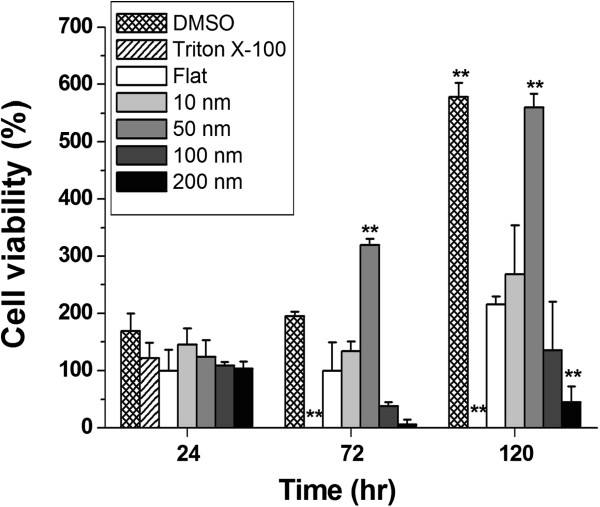
**Topographic and temporal modulation of the viability of C6 glioma cells grown on nanodot arrays.** C6 glioma cells are seeded on nanodot arrays with dot diameter ranging from 10 to 200 nm and incubated for periods of 24, 72, and 120 h. Cell viability is obtained by MTS assay. Maximum viability occurs when cells are grown on 50-nm nanodots and incubated for 72 or 120 h. Minimum growth occurs for cells seeded on 200-nm nanodot array. The DMSO-treated group (0.05 mM) serves as the positive control, while the Triton X-100-treated group (0.1% *v*/*v*) serves as the negative control. The results are expressed as the means ± standard deviation. **P < 0.01.

The C6 glioma cells were cultured on the topographical patterns and incubated for 24, 72, and 120 h. An MTS assay was performed to quantify the cell viability. The results showed no significant difference in all groups at 24 h of incubation. However, the 50-nm nanodots showed threefold viability compared to that on a flat surface at 72 and 120 h of incubation, while the cells on 100- and 200-nm nanodots showed 75% and 90% viability, respectively (Figure 1). DMSO- and Triton X-100-treated groups served as positive and negative controls, respectively.

### Cell syncytium was regulated by nanotopography

**Figure 2:**
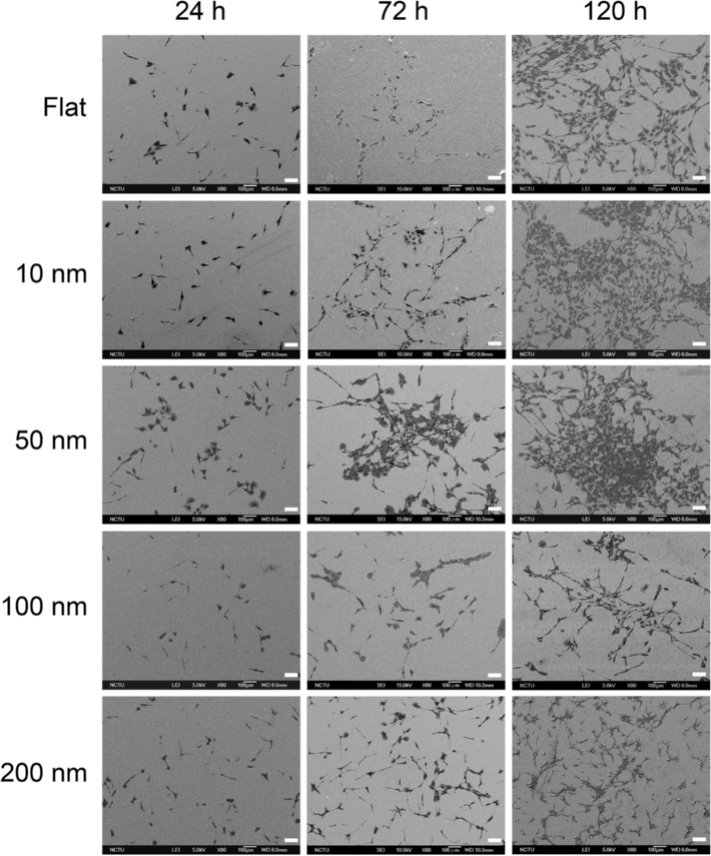
**Topographic effects on the density of branching points and meshes.** SEM images of C6 glioma cells grown on nanodot arrays. The astrocytic syncytium is fully developed at 120 h of incubation. Scale bar = 100 μm.

**Figure 3:**
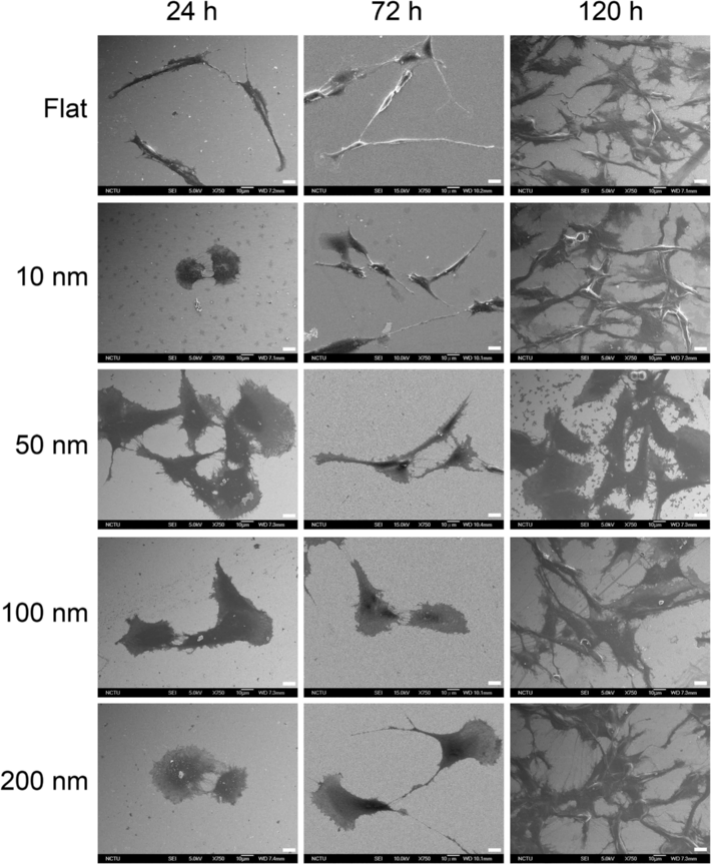
**Topographic effect on the density of branching points and meshes.** SEM images of C6 glioma cells grown on nanodot arrays showing the density of the mesh of the syncytium. Scale bar = 100 μm.

**Figure 4:**
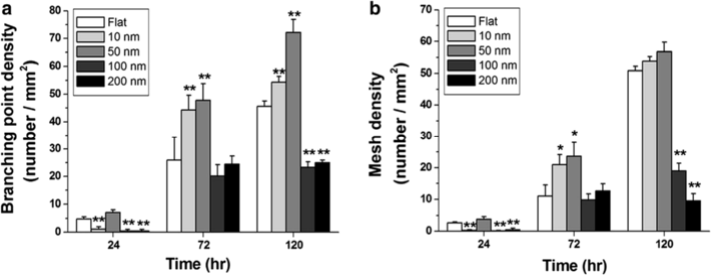
**Topographic effects on the density of branching points and meshes. (a)** The density of branching is plotted against the diameter of the nanodots and grouped by incubation time. **(b)** The density of the meshes is plotted against the diameter of the nanodots and grouped by incubation time. The values are expressed as the mean ± SD calculated from at least six experiments. *p < 0.05, **p < 0.01.

The cell morphology and astrocyte syncytium showed size dependency. The density of branching points (BPs) and mesh numbers was used to evaluate the astrocyte syncytium. The density of astrocyte BPs was defined as the number of nodes per millimeter square where different cells met (Figures 2 and 3). The cell syncytium showed maximum complexity for cells grown on 50-nm nanodots for 72 h, while 100- and 200-nm nanodot-treated groups showed less complicated growth for 72- and 120-h incubation periods. The BP density significantly increased for the 10- and 50-nm groups at 72 and 120 h (Figure 4a). However, the BP density decreased in the 100- and 200-nm nanodot-treated groups at 120 h.

Cell meshes were defined as the density of internal holes separated by cell clusters. The cell meshes became apparent at 24 h of incubation (Figure 3). C6 astrocytes seeded on 50-nm nanodots exhibited maximum cell surface area and cell syncytium, while the cells grown on 100- and 200-nm nanodots showed significant reductions in cell syncytium (Figure 4b). Clustered and well-defined cell syncytia appeared significantly at 120 h. The mesh density for 10- and 50-nm nanodot-treated groups increased at 72 h, while a significant decrease was observed for 100- and 200-nm nanodot-treated groups at 120 h.

### Nanotopography modulated astrocyte-astrocyte communication

**Figure 5:**
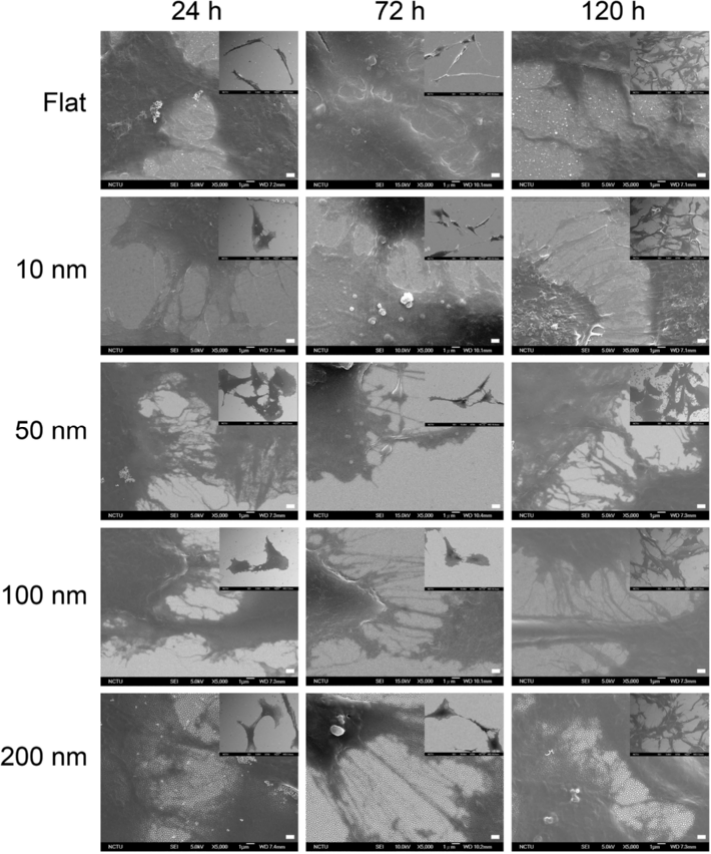
**Expanded SEM images of C6 glioma cells grown on nanodot arrays showing processes extruding from cells.** Scale bar = 1 μm. Insets are the original SEM pictures. The squares in the insets are expanded to show the processes in cell networks. Scale bar =1 μm.

Nanotopography modulated astrocyte-astrocyte interactions. Astrocytes interact with neighboring cells via astrocytic processes originating from the cell body. Topographic effects on astrocyte-astrocyte interaction are reflected in the astrocytic process number and the branching process order. The cells seeded on 50- and 100-nm nanodots exhibited more processes and higher branching order at 24, 72, and 120 h of incubation, as shown in the SEM images (Figure 5). Based on the density of BPs, the mesh orders, and the morphology of the processes, the nanotopography modulated and promoted cell syncytium formation. In addition to surface chemistry, nanotopography plays an important role in astrocytic syncytium formation.

### Nanotopography modulated the cytoskeletons, cell adhesion, and astrocytic processes of C6 glioma cells

**Figure 6:**
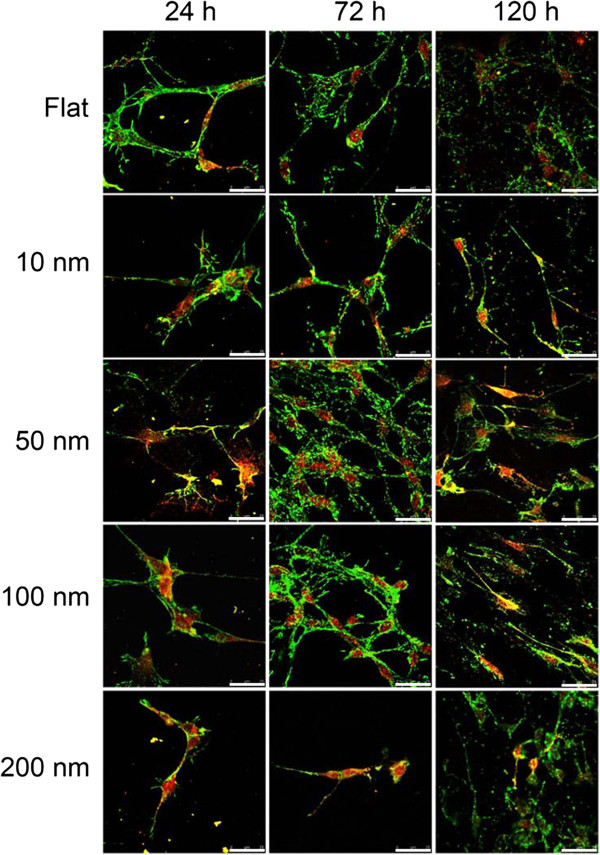
**Immunostaining of vinculin (green) and GFAP (red) in C6 glioma cells.** The cells are seeded on nanodot arrays and incubated for 24, 72, and 120 h. Images are obtained using a confocal microscope. The scale bars indicate 25 μm.

**Figure 7:**
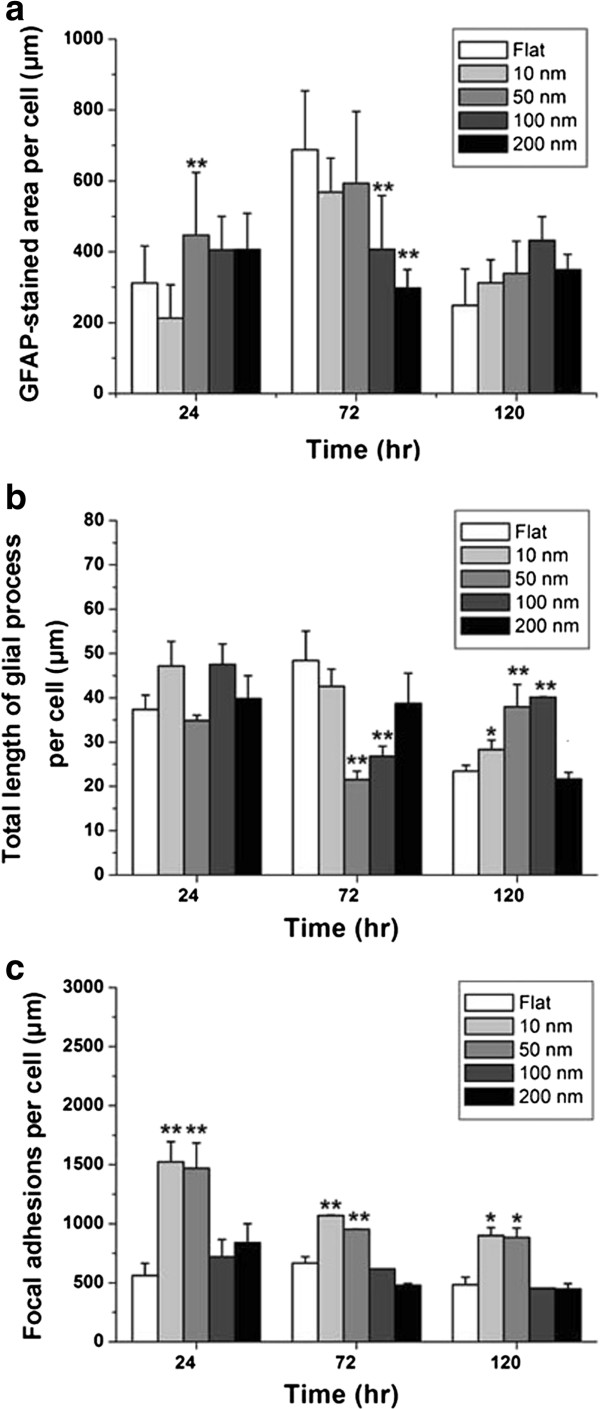
**The GFAP-stained area, total length of glial processes, and the vinculin-stained area. (a)** The GFAP-stained area per cell is plotted against the nanodot diameters and grouped by incubation time. **(b)** Total length of glial processes per cell is plotted against the nanodot diameters and grouped by incubation time. Maximum process length occurs when cells are grown on 50-nm nanodots with 120 h of incubation. **(c)** The vinculin-stained area per cell is plotted against the nanodot diameters and grouped by incubation time. Maximum staining occurs for cells grown on 10- and 50-nm nanodots. All values are expressed as the mean ± SD averaged from at least six experiments. **p < 0.01, *p < 0.01.

The cytoskeleton and astrocytic processes play important roles in the astrocytic syncytium. GFAP is an intermediate filament protein that is uniquely expressed by astrocytes [17]. GFAP initially appeared at 72 h for cells grown on 50-nm nanodots (Figures 6 and 7a). Decrease of GFAP expression was observed in cells grown on 100- and 200-nm nanodots for 72 h (Figure 7a). The effects of topography on the astrocytic processes were also observed. The 10-, 50-, and 100-nm nanodots induced longer astrocytic processes after 120 h of incubation (Figure 7b).

Vinculin is a membrane cytoskeletal protein associated with focal adhesion plaques that is involved in the linkage of integrin adhesion molecules to actin filaments [18]. The area of focal vinculin plaques significantly increased in the 10- and 50-nm nanodot-treated groups at 24, 72, and 120 h (Figure 7c).

### Nanotopography enhanced connexin43 transport

**Figure 8:**
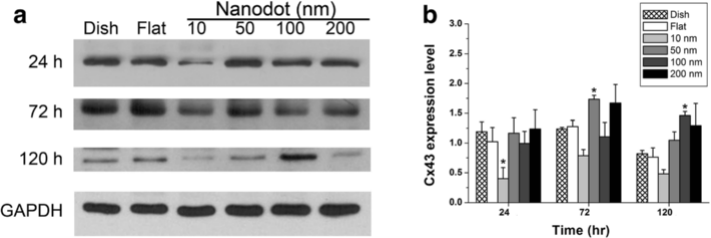
**Quantitation of connexin43 expression in C6 glioma cells grown on nanodot arrays. (a)** Western blotting of C6 glioma cells with anti-Cx43 antibody. GAPDH staining serves as a control. **(b)** Expression of Cx43 relative to GAPDH is plotted against the nanodot diameters and grouped by incubation time. Values are expressed as the mean ± SD averaged from at least three independent experiments. *p < 0.05.

Nanodot arrays control astrocyte-astrocyte interaction by regulating the function of gap junction proteins. Cx43, which composes gap junction channels (GJCs), mediates transmission and dispersion growth/suppressive factors and reveals the contact spots between astrocytes [19,20]. The expression level of Cx43 did not show a consistent pattern regarding the dot diameter (Figure 8). The 10-nm nanodots decreased the expression of Cx43 at 24 h. The Cx43 expression level significantly increased for cells grown on 50-nm nanodots for 72 h.

### Nanotopography modulated the expression and transport of Cx43 protein

**Figure 9:**
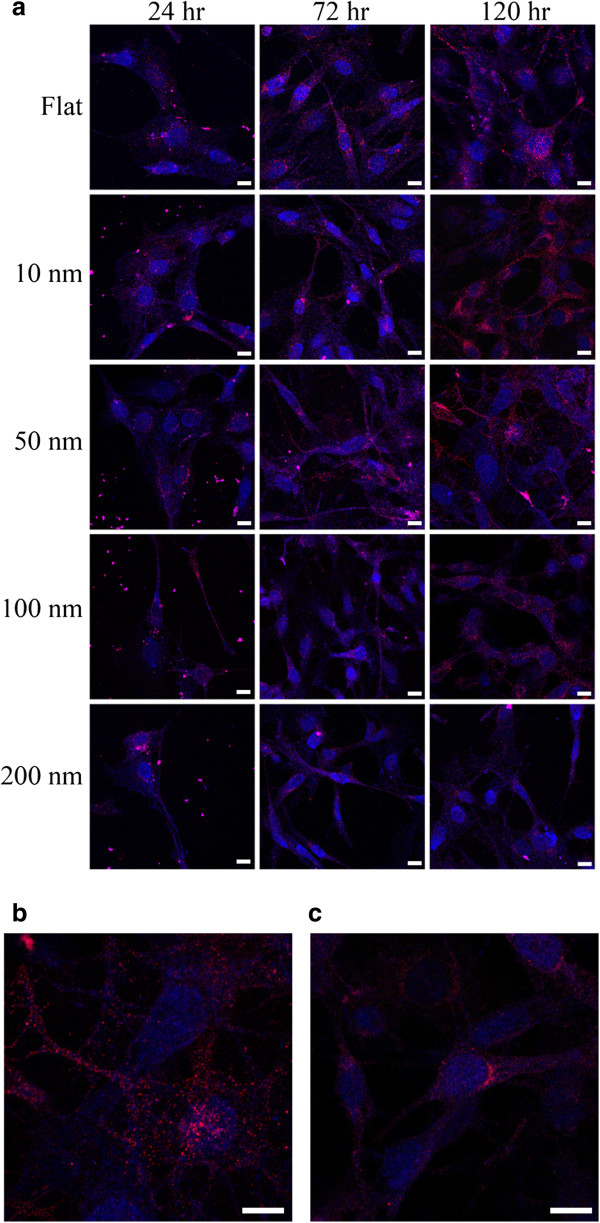
**Immunostaining and enlarged images showing localized and spread of Cx43 protein expression. (a)** Time-dependent immunostaining of GFAP (blue) and connexin43 (red) in C6 glioma cells grown on nanodot arrays. Enhanced expression of Cx43 occurs to 10 and 39 nm at 120 h of incubation. **(b)** Enlarged image showing reduced and nucleus-localized expression of Cx43 protein in C6 glioma cells grown on 100-nm nanodots. **(c)** Enlarged image showing extensive expression of Cx43 protein spread throughout the entire cell. Scale bar = 5 μm.

Immunostaining was used to obtain the expression and cellular localization of Cx43 in C6 glioma cells on nanodot arrays. The Cx43 immunoreactivity plaques were displayed intensely in the nuclei of cells grown on 10-, 50-, and 100-nm nanodots for 24 h (Figure 9a). After 72 h, Cx43 was located at the astrocytic processes in the control group and in the 10- and 50-nm nanodot-treated groups, while Cx43 remained in the nuclei for the 100- and 200-nm nanodot-treated groups. After 72 h, Cx43 accumulated preferentially at the astrocytic processes and boundaries for cells grown on 10- and 50-nm nanodots (Figure 9b). Cx43 was located throughout the cells from the nuclei to the processes for 100- and 200-nm treated groups (Figure 9c). The results suggest that the nanotopography modulated the expression level and cellular transport of Cx43 protein in C6 glioma cells.

Nanostructured surfaces provide tunable environments on which to culture neural cells for investigating cell-matrix interactions [2,21]. Here, we provide evidence that nanodot surfaces, ranging from 10 to 200 nm, were capable of modulating neuronal interaction and communication.

Enhancing the viability and adhesion of glial cells leads to favorable neuronal physiological functions. Mitomycin C and retinoic acid (RA) have been shown to inhibit cell proliferation and induce morphological changes in C6 cells [22,23], but the ability of materials to improve C6 growth is less well known. Maximum cell proliferation occurred on the 50-nm nanodot surface, which was approximately twofold greater than that on flat surfaces. On the other hand, astrocytes have good spreading and focal adhesions when grown suspended in a manner corresponding to greater inter-pillar spacing. Focal adhesion complexes were well developed on small pillars; thus, submicron architecture is important for proper focal adhesion formation [2]. Our results indicated that 10- and 50-nm nanodots enhanced cell attachment, whereas 100- and 200-nm nanodot arrays reduced the formation of focal adhesions.

Astrocytes play a powerful role in setting up the basic scaffolding of the brain during development. By interacting with cell adhesion molecules on the glial membrane, neurons migrate along the appropriate glial processes and extend axons and dendrites following the guidance of the glia to form proper synaptic connections [1]. Proper synaptic contacts between axons (neurons) and processes (astrocytes) indicate beneficial neuronal physiological functions. Our results showed that proper network formation was significantly increased for cells grown on 10- and 50-nm nanodot surfaces. However, astrocytes seeded on 100- and 200-nm nanodots showed reduced branch point and mesh numbers.

Cx43 regulates cell-cell interactions in the nervous system. Tetrodotoxin reduced the Cx43 immunoreactivity in the hippocampal nervous system in mice [24]. Mg^2+^-picrotoxin increased the Cx43 expression level [3]. The effects of controlling Cx43 expression and transport with nanostructures are unclear. Based on our results, Cx43 expression levels were increased on 10- and 50-nm nanodots compared to those in other groups. The transport of Cx43 was accelerated from the nuclei to the processes on 10- and 50-nm nanodots compared to 100- and 200-nm nanodots. Nanotopography effectively controls the expression and transport of signal transduction proteins in astrocytes.

Nanopatterns are used basic neurobiology in tissue-engineered scaffolds [25-27], nerve prostheses [28], and neurobiosensors [13,29]. The current study provides further evidence that nanotopography regulates cell-cell interactions and communication by controlling the cell growth and gap junction proteins. Astrocytic networking may be controlled by size-dependent regulation, and the optimal microenvironment could support ideal neuronal regeneration and function. Nanopatterned scaffolds stimulate astrocytes and regulate glia-glia interactions. The results of this study show that nanodot arrays directed the growth of and promoted communication in astrocytic networks. We demonstrated that nanodots regulate the physiology, signaling transduction, and cell-cell interaction of glial cells. Furthermore, controlling neuronal physiological behavior with optimized nanosurfaces could be exploited to develop biocompatible devices in the nervous system.

## Conclusions

The nano-scale cell-substrate interaction regulates glia-glia communication. The results of this study showed that nanodot arrays effectively regulate the viability, morphology, cytoskeleton, adhesion, and astrocytic syncytium of C6 astroglia. The 50-nm nanodots especially enhanced cell growth. The expression of Cx43 was significantly enhanced and transported to the processes for cells grown on the 10- and 50-nm nanodot surfaces. Nanotopography not only regulated the expression but also enhanced the transportation for proteins associated with cell-cell networking. By fine-tuning nanotopography, it is possible to modulate the physiological behavior of astrocytes and optimize neuronal interactions, including neuronal hyperexcitability and epileptic activity. This is specifically useful to improve implantable neuroprosthetic devices or neuron regeneration therapies.

## Competing interests

The authors declare that they have no competing interests.

## Authors’ contributions

CHL and GSH are responsible for the concept and design of the study. GSH, CHL, and YWC prepared the manuscript. CHL and YWC performed the experiments and data analysis. All authors read and approved the final manuscript.

## Authors’ information

GSH received his BS degree in Chemical Engineering from NCTU, Taiwan. He joined the PhD program of Biochemistry and Molecular Biology at Hershey Medical Center, Penn State University and received his PhD degree. He soon studied Structural Biology at Terrence Oas's lab as a postdoctoral fellow. In 2003, he became the first faculty at the Institute of Nanotechnology NCTU and served as Chairman from 2007 to 2009. His current research focuses in the protein-based molecular devices and their application. CHL received her masters degree in nanotechnology from National Chiao Tung University (NCTU) in 2010. She is currently a candidate for doctor's degree in materials science and engineering at NCTU. She was a teaching assistant with Hui Liang Wang group in National Kaohsiung Normal University from 2006 to 2008. She has been a research assistant with the G. Steve Huang group in NCTU, where she studied the interaction and application of biology and nanotechnology interface, since 2008. Her current research interests include aging, cell signaling, bioelectronics, and the bio-nano interaction. She has presented two papers on nanoparticle neurotoxicity at international conference in Taiwan and Japan. She has polished three articles on magnetic field inducing aging in *Caenorhabditis elegans*. YWC received her masters degree in nanotechnology from NCTU in 2013. Her research interests include bioelectronics and the bio-nano interaction.
